# Initial Response to Antiepileptic Drugs in Patients with Newly Diagnosed Epilepsy As a Predictor of Long-term Outcome

**DOI:** 10.3389/fneur.2017.00658

**Published:** 2017-12-08

**Authors:** Lu Xia, Shuchun Ou, Songqing Pan

**Affiliations:** ^1^Department of Neurology, Renmin Hospital of Wuhan University, Wuhan, China

**Keywords:** antiepileptic drugs, early response, long-term outcome, brain-imaging abnormalities, pretreatment seizure numbers

## Abstract

**Objective:**

To investigate the correlation between initial response to antiepileptic drugs (AEDs) and long-term outcomes after 3 years in patients with newly diagnosed epilepsy.

**Methods:**

This prospective study included 204 patients with newly diagnosed epilepsy, who were followed-up for at least 36 months. The long-term seizure freedom at 36 months (36MSF) was evaluated in patients with seizure freedom 6 months (6MSF) or 12 months (12MSF) after initial treatment vs those with no seizure freedom after the initial 6 months (6MNSF) or 12 months (12MNSF). Univariate analysis and a multiple logistic regression model were used to analyze the association of potential confounding variables with the initial response to AEDs.

**Results:**

The number of patients with 36MSF was significantly higher for patients that had 6MSF (94/131, 71.8%) than those that had 6MNSF [16/73, 21.9%; χ^2^ = 46.862, *p* < 0.0001, odd ratio (OR) = 9.051]. The number of patients with 36MSF was significantly higher in patients that had 12MSF (94/118 79.7%) than those that had 12MNSF (19/86, 22.1%; χ^2^ = 66.720, *p* < 0.0001, OR = 13.811). The numbers of patients that had 36MSF were not significantly different between patients that experienced 6MSF and 12MSF or between patients that had 6MNSF and 12MNSF. Abnormalities observed in magnetic resonance imaging or computed tomography and the number of seizures before treatment correlated with poor initial 6-month response to AEDs.

**Significance:**

The initial 6-month response to AEDs is a valuable predictor of long-term response in patients with newly diagnosed epilepsy. The number of seizures before treatment and brain-imaging abnormalities are two prognostic predictors of initial 6-month seizure freedom.

## Introduction

Epilepsy is one of the most common chronic brain diseases, affecting more than 50 million people worldwide (1). Although antiepileptic drugs (AEDs) can effectively control seizures in approximately 60–70% of patients with epilepsy, approximately 30% of patients with partial epilepsy and 25% of patients with generalized epilepsy have refractory seizures that are difficult to manage (2–4). Therefore, early assessment of long-term therapeutic benefit is essential for clinical practice and patient counseling, or early referral for epilepsy surgery (5–7).

Previous studies have found that early response to AEDs is related to long-term seizure freedom (6, 8–10). Schmidt (11) reported that patients who are seizure-free for the initial 6 months have a 90% chance of being seizure-free at 12 months, whereas those who are not seizure-free at 6 months only have a 45% chance of being seizure-free at 12 months. This suggests that the response to AEDs in the initial 6 months is a good predictive indicator for the longer-term 12-month outcome. In a cohort of 107 patients with newly diagnosed epilepsy, Lindsten et al. (12) reported that all patients who were seizure-free 1 year after AED treatment achieved 5-year remission and only 34% of patients who had more than one seizure 1 year after diagnosis achieved 5-year remission. Accordingly, they suggested that seizure freedom 1 year after AED treatment was a good predictor of long-term remission. Although an association between the early response to AEDs over the initial 6 or 12 months with long-term outcomes in patients with newly diagnosed epilepsy has been reported, no observational studies have been performed that compare the prognostic value of the initial seizure freedom at 6 vs 12 months after AED treatment in the prediction of long-term seizure freedom.

In this study, we conducted a hospital-based study in patients with newly diagnosed epilepsy, who were followed-up for more than 3 years after AED treatment. The purpose of this study was to investigate if initial seizure freedom at 6 months can be used as an early predictor of long-term prognosis after 3 years, and to identify clinical variables that are associated with initial response to AEDs.

## Materials and Methods

### Study Subjects

The study was approved by our institutional review board, and all subjects gave their informed consent. This prospective study included a total of 1,570 consecutive patients with newly diagnosed epilepsy, who visited the Epilepsy Outpatient Clinic at the Renmin Hospital of Wuhan University (Hubei, China) from June 1, 2009 to December 30, 2015. The inclusion criteria were as follows: patients with (1) newly diagnosed epilepsy; (2) a history of two or more clinically definite unprovoked seizures occurring at least 24 h apart, or evidence of a prior brain lesion resulting in seizure, or electroencephalography (EEG) epileptiform abnormalities and a significant brain-imaging structural abnormality, if they had only one seizure (13). The exclusion criteria were as follows: (1) patients with chronic epilepsy; (2) poor compliance; (3) patients lost to follow-up; (4) patients with a follow-up period of less than 3 years; (5) patients with onset interval of over 6 months; and (6) patients with progressive pathology, such as brain tumors, and epileptic encephalopathy. The diagnosis and evaluation were made by three experienced epileptic experts. Finally, 204 of 1,570 patients met the criteria and were included in the study.

The following information was recorded during the first visit: sex, age at seizure onset, pretreatment seizure numbers, pretreatment duration, epilepsy etiology, seizure type, EEG and magnetic resonance imaging (MRI) findings, family history of epilepsy, and history of febrile seizure. Epilepsy and seizure were classified according to the proposal of the International League Against Epilepsy (ILAE) (14–17). The seizure types were divided into generalized seizure and partial seizure. Epilepsy was classified as idiopathic and symptomatic seizure based on the etiology. The AEDs used in our Epilepsy center included valproate (VPA), carbamazepine (CBZ), oxcarbazepine (OXC), lamotrigine (LTG), topiramate (TPM), levetiracetam (LEV), and clonazepam (CZP). According to ILAE and National Institute for Health and Care Excellence guidelines, the study was treated with CBZ, LTG, VPA, and LEV for adults with partial-onset seizures, OXC for children with partial-onset seizures, LTG and VPA for adults with generalized-onset tonic–clonic seizures, and VPA for children with generalized-onset tonic–clonic seizures as first-line options (18–20). Monotherapy with AEDs was used as the first-line treatment of choice. We start with low-dose at first and increase based on efficacy and tolerability but not exceed the limit dose (2,000 mg for VPA, 1,000 mg for CBZ, 1,500 mg for OXC, 250 mg for LTG, 300 mg for TPM, 2,000 mg for LEV, and 6 mg for CZP). If the first AED proved to be inefficient at enough dosage, an alternative AED was used as a substitute or added according to each patient’s condition. A combination of three AEDs or more was avoided. AEDs were withdrawn and substituted immediately if serious side effects occurred. All patients were followed-up for more than 3 years through clinic visits or telephone calls. During the follow-up, the presence or absence of seizures and drug regimens were recorded.

### Study Design

In this study, we evaluated the prognostic value of early therapeutic response to AEDs for the long-term outcome in patients with newly diagnosed epilepsy. Early and long-term responses to AEDs were defined as the absence of seizure for 6 and 36 months, respectively. The individual response evolution was defined as the change in response to AEDs at 6 and 36 months. We compared the response evolution in patients who were initially seizure-free at 6 months (6MSF) or at 12 months (12MSF) and had no seizure for 36 months with those who were not initially seizure-free at 6 months (6MNSF) or at 12 months (12MNSF) but had no seizure thereafter. Patients with seizures that occurred during the titration phase were excluded.

In addition, we also analyzed the influence of factors such as patient sex, age at seizure onset, pretreatment duration, seizure numbers before treatment, epilepsy etiology, seizure type, family history of epilepsy, history of febrile seizure, epileptiform discharges on EEG, and the presence of structural lesions on MRI or computed tomography (CT) in the initial 6-month response to AEDs.

### Statistical Analysis

Statistical analyses were performed using SPSS 22.0 (SPSS Inc., IL, USA) and GraphPad Prism 7.0 software. Chi-squared tests were used to compare differences in the long-term outcomes between patients with 6MSF vs 6MNSF, 12MSF vs 12MNSF. As the groups of 6MSF and 12MSF, 6MNSF and 12MNSF are not independent or mutually exclusive, when we longitudinally compare the differences of long-term seizure remission between patients with 6MSF vs 12MSF and 6MNSF vs 12MNSF, customary statistical tests were unsuited. For comparing the differences, we assessed the proportions of long-term remission patients within 6MSF vs 12MSF and 6MNSF vs 12MNSF. Uncertainly of the estimates was controlled for by using modified Wald method 95% confidence intervals (CIs) form the binomial distribution (21). The rates and their CIs are presented as a forest plot. If the 95% CIs of the estimates were not overlapping, the seizure freedom rates were considered to be distinct between the categories (22). Each potential confounding variable was analyzed in patients with 6MSF vs 6MNSF with Chi-squared tests for Univariate analysis. Multiple logistic regression was used to analyze the prognostic predictors with significant difference on univariate analysis. Kaplan–Meier survival analysis was used to assess the time until the first seizure recurrence during maintenance treatment periods in different groups. A *p* value < 0.05 was considered statistically significant.

## Results

### Clinical Characteristics

A total of 204 patients (80 females and 124 males) were included in this study. Table 1 summarizes the detailed clinical characteristics of the 204 patients. The average age at onset of epilepsy was 17.0 years (range, 2–55 years). The mean follow-up duration was 4.8 years (range 3–6.5 years). Most patients (52.5%) had pretreatment over 6 months. Most patients (35.3%) started AED treatment after two unprovoked seizures, and only seven patients started AED treatment after the first unprovoked seizure. Epileptiform abnormalities on EEG were observed in 165 (80.9%) patients. Abnormal brain imagines were observed in 72 (35.3%) patients, including 14 patients with dysplasia, 18 patients with demyelination, 7 patients with hippocampal sclerosis, and 33 patients with posttraumatic damage.

**Table 1:** Clinical characteristics of the 204 patients.

	*N*	%
**Gender**		
Women	80	39.2
Men	124	60.8
**Age at seizure onset (years)**		
≤16	115	56.4
>16	89	43.6
**Number of seizures before treatment**		
1–9 times	166	81.4
≥10 times	38	18.6
**Pretreatment duration (months)**		
<6	97	47.5
≥6	107	52.5
**Seizure type**		
Partial	157	77.0
Generalized	47	23.0
**Epilepsy etiology**		
Idiopathic	74	36.3
Symptomatic	130	63.7
**MRI or CT record at entry**		
Normal	132	64.7
Abnormal	72	35.3
**EEG at entry**		
Normal	39	19.1
Abnormal	165	80.9
**Family history**		
No	195	95.6
Yes	9	4.4
**History of febrile seizure**		
No	188	92.2
Yes	16	7.8

*6MSF, patients who were seizure-free over the initial 6 months; 6MNSF, patients who were not seizure-free over the initial 6 months; EEG, electroencephalography; MRI, magnetic resonance imaging; CT, computed tomography*.

### The Response Evolution to AEDs

**Figure 1:**
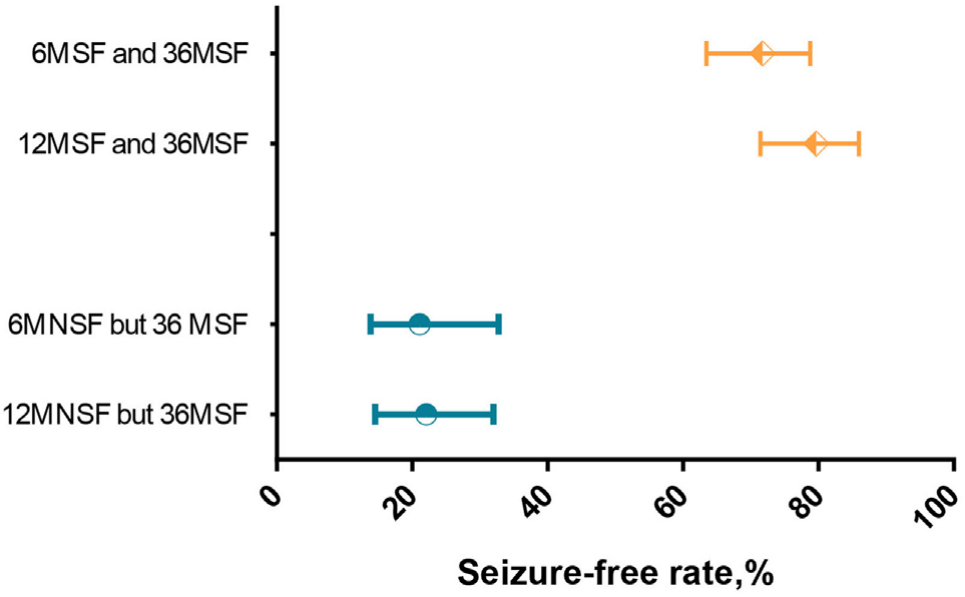
Forest plot of long-term seizure freedom rates with modified Wald method 95% confidence interval (CI) for patients with 6MSF vs 12MSF (orange) and 6MNSF vs 12MNSF (blue). Overlapping CIs indicate no different long-term seizure freedom rates between 6MSF vs 12MSF and 6MNSF vs 12MNSF.

Of the 204 patients, 131 (64.2%) patients were seizure-free over the initial 6 months (6MSF) after AED initiation. Of the 131 patients with 6MSF, 94 (71.8%) were seizure-free for up to 36 months (36MSF) and 37 (28.2%) patients had at least one seizure over 7–36 months (36MNSF). By contrast, of the 73 (73/204, 35.8%) patients who were not seizure-free over the initial 6 months (6MNSF), only 16 (16/73, 21.9%) patients were seizure-free from 7–36 months and 57 (57/73, 78.1%) patients were not seizure-free during the whole study period. The number of patients with 36MSF was significantly higher in patients with 6MSF compared to those with 6MNSF [χ^2^ = 46.862, *p* < 0.0001, odd ratio (OR) = 9.051]. Similarly, the number of patients with 36MSF was significantly higher in patients with 12MSF than those with 12MNSF (χ^2^ = 66.720, *p* < 0.0001, OR = 13.811) (Table 2). However, Table 3 presented the proportions of 36MSF patients with its 95% CI after initial 6MSF vs 12MSF and 6MNSF vs 12MNSF. Figure 1 presented the rate of long-term seizure freedom with modified Wald method 95% CI for patients with 6MSF vs 12MSF (orange) and 6MNSF vs 12MNSF (blue) as forest plot. Overlapping of 95% CIs means that the accuracy of the long-term seizure freedom rate estimated did not significantly differ between patients with 6MSF and 12MSF, nor was there any significant difference between patients with 6MNSF and 12MNSF.

**Table 2:** The evolution of seizure freedom after the initial response in 204 patients with newly diagnosed epilepsy.

	*N* (%)	χ^2^	*p-*Value	OR	95% CI
**All patients (***N*** = 204)**					

**Compare the evolution of seizure freedom between 6MSF (***N*** = 131) vs 6MNSF (***N*** = 73)**					
Seizure-free at 6 and 36 months	94 (71.8)	46.862	*p* < 0.0001	9.051	4.620–17.730
Not seizure-free at 6 months but seizure-free at 36 months	16 (21.9)				
**Compare the evolution of seizure freedom between 12MSF (***N*** = 118) vs 12MNSF (***N*** = 86)**					
Seizure-free at 12 and 36 months	94 (79.7)	66.720	*p* < 0.0001	13.811	7.007–27.223
Not seizure-free at 12 months but seizure-free at 36 months	19 (22.1)				

*6MSF, patients who were seizure-free over the initial 6 months; 6MNSF, patients who were not seizure-free over the initial 6 months; 12MSF, patients who were seizure-free over the initial 12 months; 12MNSF, patients who were not seizure-free over the initial 12 months; OR, odds ratio; CI, confidence interval*.

**Table 3:** Longitudinally compare the evolution of seizure freedom after early response of AEDs at 6 and 12 months in newly diagnosed epilepsy.

	*N* (%)	95% CI
**Longitudinally compare the evolution of seizure freedom between 6MSF (***N*** = 131) vs 12MSF (***N*** = 118)**		
Seizure-free at 6 and 36 months	94 (94/131, 71.8%)	63.5–78.8
Seizure-free at 12 and 36 months	94 (94/118, 79.7%)	71.5–86.0
**Longitudinally compare the evolution of seizure freedom between 6MNSF (***N*** = 73) vs 12MNSF (***N*** = 86)**		
Not seizure-free at 6 but seizure-free at 36 months	16 (16/73, 21.9%)	13.9–32.8
Not seizure-free at 12 but seizure-free at 36 months	19 (19/86, 22.1%)	14.6–32.0

*6MSF, patients who were seizure-free over the initial 6 months; 6MNSF, patients who were not seizure-free over the initial 6 months; 12MSF, patients who were seizure-free over the initial 12 months; 12MNSF, patients who were not seizure-free over the initial 12 months; CI, confidence interval*.

### The Relationship between Clinical Variables and the Initial 6-Month Response to AEDs

Univariate analysis showed that the early 6-month response to AEDs was negatively correlated with the number of seizures before treatment (*p* = 0.005). Abnormalities on MRI or CT were significantly associated with poor initial 6-month response to AEDs (*p* = 0.027). Factors such as gender (*p* = 0.313), age at seizure onset (*p* = 0.734), pretreatment duration (*p* = 0.210), seizure type (*p* = 0.328), epilepsy etiology (*p* = 0.875), EEG result at diagnosis (*p* = 0.723), family history of epilepsy (*p* = 0.579), and history of febrile seizure (*p* = 0.349) were not significantly associated with the early 6 months response to AEDs (Table 4).

**Table 4:** Patients with 6MSF (*N* = 131) vs 6MNSF (*N* = 73) as a prognostic factor.

	6MSF, *N* (%)	6MNSF, *N* (%)	*p*-Value	OR	95% CI
**Gender**					
Women	48 (36.6)	32 (43.8)	0.313	0.741	0.414–1.328
Men	83 (63.4)	41 (56.2)			
**Age at seizure onset (years)**					
≤16	75 (57.3)	40 (54.8)	0.734	1.105	0.621–1.966
>16	56 (42.7)	33 (45.2)			
**Number of seizures before treatment**					
1–9 times	114 (87.0)	52 (71.2)	0.005*	2.708	1.320–5.556
≥10 times	17 (13.0)	21 (28.8)			
**Pretreatment duration (months)**					
<6	58 (44.3)	39 (53.4)	0.210	0.693	0.390–1.231
≥6	73 (55.7)	34 (46.6)			
**Seizure type**					
Partial	98 (74.8)	59 (80.8)	0.328	0.705	0.349–1.424
Generalized	33 (25.2)	14 (19.2)			
**Epilepsy etiology**					
Idiopathic	47 (35.9)	27 (37.0)	0.875	0.953	0.526–1.727
Symptomatic	84 (64.1)	46 (63.0)			
**MRI or CT record at entry**					
Normal	92 (70.2)	40 (54.8)	0.027*	1.946	1.075–3.525
Abnormal	39 (29.8)	33 (45.2)			
**EEG at entry**					
Normal	26 (19.8)	13 (17.8)	0.723	1.143	0.547–2.389
Abnormal	105 (80.2)	60 (82.2)			
**Family history**					
No	126 (96.2)	69 (94.5)	0.579	1.461	0.380–5.619
Yes	5 (3.8)	4 (5.5)			
**History of febrile seizure**					
No	119 (90.8)	69 (94.5)	0.349	0.575	0.178–1.852
Yes	12 (9.2)	4 (5.5)			

*6MSF, patients who were seizure-free over the initial 6 months; 6MNSF, patients who were not seizure-free over the initial 6 months; EEG, electroencephalography; MRI, magnetic resonance imaging; CT, computed tomography; OR, odds ratio; CI, confidence interval*.

*p-Values obtained from chi-square tests with significant statistical differences.

Multiple logistic regression was used to analyze the prognostic predictors with significant difference on univariate analysis. Therefore, we add the variables of the number of seizures before treatment and the brain-imaging results in the multivariate logistic regression analysis by backward way. In multivariate logistic regression analysis, the number of seizures before treatment and the brain-imaging results remained significantly different distributions in the 6MSF and 6MNSF groups. The OR of poor initial 6-month response to AEDs was 2.671 (95% CI 1.423–5.013) in patients with 10 or more seizures before treatment. The number of patients that reached 6 months seizure-free was significantly lower in patients that had 10 or more seizures before treatment than those that suffered only 1–9 seizures before treatment (*p* = 0.002). The OR of poor initial 6-month response to AEDs was 1.919 (95% CI 1.158–3.180) in patients presenting with brain-imaging (MRI or CT) abnormalities (Table 5).

**Table 5:** Multivariate logistic regression analysis to explore the clinical variables of not being seizure-free at initial 6 months.

Clinical variables	OR	95% CI	*p*-Value
Abnormal MRI or CT result	1.919	1.158–3.180	0.011
≥10 seizures before treatment	2.671	1.423–5.013	0.002

*MRI, magnetic resonance imaging; CT, computed tomography; OR, odds ratio; CI, confidence interval*.

**Figure 2:**
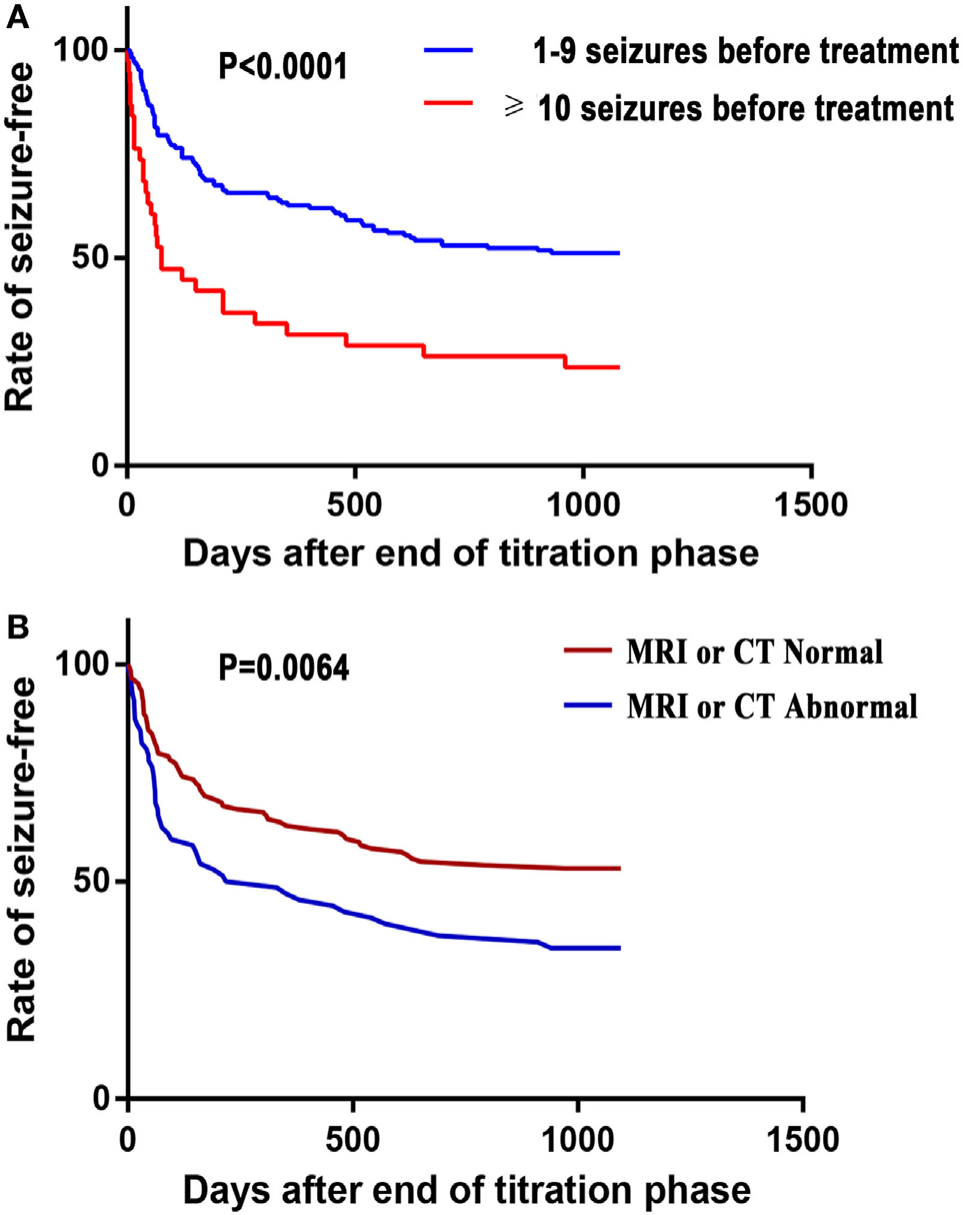
Kaplan–Meier survival analysis of the time until the first seizure recurrence during antiepileptic drug treatment against the number of seizures before treatment **(A)** and brain-imaging results **(B)**.

In Kaplan–Meier survival analysis, first seizure recurrence during AED treatment was significantly earlier among patients that had 10 or more seizures before treatment compared with those that had suffered 1–9 seizures before treatment (*p* < 0.0001; Figure 2A). The time until the first seizure was significantly different in patients with MRI or CT abnormalities than those without during AED treatment (*p* < 0.0064, Figure 2B).

## Discussion

In this study, the two main conclusions were as follows: (1) Response to AEDs over the initial 6 months serves as a good predictor of 36-month long-term outcome in patients with newly diagnosed epilepsy. It is not necessary to extend to 12 months for predicting the long-term outcome. Patients that responded poorly to the initial AED treatment are less likely to be seizure-free in the long run. (2) Patients with 10 or more seizures before treatment and with brain-imaging (MRI or CT) abnormalities were associated with poor initial 6-month response to AEDs.

In 2006 and 2013, the ILAE recommended that 12 months of remission, or three times the longest pretreatment inter-seizure interval, should be used as the minimum period to evaluate the long-term effectiveness of AEDs. Moreover, the recommended minimum period to assess the efficacy of AEDs is seizure freedom of 6 months (18, 20). In this study, we chose 36 months to evaluate the long-term effectiveness of AEDs, which, we believe, better reflects the long-term outcome of patients with newly diagnosed epilepsy compared with the 12 months used by most studies (11, 23, 24). It has been reported that 74.9% patients with newly diagnosed epilepsy were seizure-free over the first 6 months after starting AED treatment, and remained seizure-free for at least 12 months on unchanged treatment (23). Schmidt found that patients with seizure freedom over the initial 6 months had a 90% chance of being seizure-free at 12 months (11). In this study, we found that patients who were seizure-free over the initial 6 months had a 71.8% chance of being seizure-free at 36 months, whereas patients who were not seizure-free over the initial 6-month period had only a 21.9% chance of being seizure-free by 36 months. Furthermore, we found that the number of patients who were seizure-free at 36 months was not significantly different between patients who were seizure-free over the initial 6 or 12 months (71.8 vs 79.7%). Our findings support the theory that early response to AEDs over the initial 6 months is not only a powerful indicator of 12-month prognosis but is also an excellent predictor of the 3-year outcome for patients with newly diagnosed epilepsy. Notably, it is unnecessary to extend to 12 months for predicting the long-term outcome. In addition, since only 21.9% (16/73) of patients who failed to respond to AEDs were seizure-free after 36 months, early evaluation and identification of refractory epilepsy may be important for these patients to select nondrug therapies such as surgery, ketogenic diet and vagus-nerve stimulation.

Several studies have demonstrated that a high number of seizures before treatment is associated with poor response to AEDs (2, 25–28). Consistent with these studies, we found that 87.0% (114/131) of patients who suffered 1–9 seizures before AED treatment were seizure-free within the initial 6 months of treatment, while seizure freedom over the initial 6 months was 13.0% (17/131) for patients who experienced 10 or more seizures before treatment, respectively. 10 or more seizure occurrences were a significant predictor of poor response to early AEDs. This may be due to pathological conditions in the hippocampus, in which neuronal loss and mossy fiber sprouting are triggered by repeated seizures, leading to the formation of excitatory recurrent circuits (29). However, several studies have found that immediate AED treatment after the first unprovoked seizure appeared to reduce the risk of short-term recurrence, but did not improve the long-term prognoses (13, 30–32). Moreover, it has been reported that an increased number of seizures prior to AED treatment may be the result of pathophysiologic epilepsy changes, which may manifest as drug refractoriness, but do not cause drug refractoriness (2, 33, 34). The specific mechanisms that underpin drug refractoriness are still poorly understood, and warrant further study.

In this study, we found that brain-imaging abnormalities were associated with poor long-term outcomes in patients with newly diagnosed epilepsy, which is consistent with previous studies (13, 35–37). According to the 2015 ILAE evidence-based guideline about the management of an unprovoked first seizure in adults, significant brain-imaging abnormalities (Level B) are associated with increased risk of seizure recurrence (13). Arthur et al. reported that MRI abnormalities were associated with increased risk of seizure recurrence only over the initial 9 months, but not over 18–27 months, in 150 children with normal physical and neurological examination results (37). In this study, we found that patients with brain-imaging abnormalities were less likely to reach 6-month seizure freedom. Furthermore, the first seizure recurrence was significantly earlier in patients that presented with brain-imaging abnormalities than those with normal MRI or CT records at entry. Therefore, examinations such as MRI or CT should be used as routine tests for newly diagnosed epilepsy. MRI and CT are not only used to assess the seizure outcome for patients with newly diagnosed epilepsy but also valuable for identifying other neurological disorders such as hippocampal sclerosis, focal cortical dysplasia, and brain tumors, which can be treated with surgery.

Several factors have been reported to be associated with a favorable outcome in patients with newly diagnosed epilepsy, including shorter duration of epilepsy, no epileptiform discharges, late age at seizure onset, and idiopathic epilepsy (8, 26, 29, 38–40). By contrast, our study found that only the number of seizures before treatment and brain-imaging abnormalities were associated with the early response to AEDs. Differences in population and design may be responsible for disparities among studies.

There are some limitations to the present study. First, as an observational study, our study is unable to illustrate the reason why early response to AEDs was significantly correlated with long-term outcome in patients with newly diagnosed epilepsy. Second, the sample size of our cohorts is relatively small. It is possible that some prognosis factors may be missed due to the small sample size. Further studies with a larger sample cohort are required.

To summarize, we found that the response to AEDs over the initial 6 months is a good predictor for evaluating long-term response in patients with newly diagnosed epilepsy. Our study suggests that patients with refractory epilepsy at the onset will also be refractory to AEDs with treatment. Our findings support the view that response to AEDs reflects inherent disease severity that is influenced by underlying pathology and genetics. Patients with more severe disease are more likely to have a higher number of seizures at the time of diagnosis. Patients with abnormal brain imaging have less probability of long-term remission. It is important to elucidate the pathogenesis of epilepsy, which may help to identify new treatments to cure the epilepsy itself, not just the seizures, and to devise alternative therapeutic strategies should AED treatment fail.

## Ethics Statement

This study was carried out in accordance with the recommendations of “Ethics Review Committee of Wuhan University Renmin Hospital” with written informed consent from all subjects. All subjects gave written informed consent in accordance with the Declaration of Helsinki. The protocol was approved by the “Ethics Review Committee of Wuhan University Renmin Hospital.”

## Author Contributions

LX: study design and draft the work; SO: picture editing and date analysis; SP: revising it critically for important intellectual content. All the authors read and approved the final manuscript.

## Conflict of Interest Statement

The authors declare that the research was conducted in the absence of any commercial or financial relationships that could be construed as a potential conflict of interest.
